# Early postnatal rat ventricle resection leads to long‐term preserved cardiac function despite tissue hypoperfusion

**DOI:** 10.14814/phy2.12115

**Published:** 2014-08-28

**Authors:** Camila Zogbi, Ana E. T. Saturi de Carvalho, Juliana S. Nakamuta, Viviane de M. Caceres, Silvana Prando, Maria C. P. Giorgi, Carlos E. Rochitte, Jose C. Meneghetti, Jose E. Krieger

**Affiliations:** 1 Heart Institute (InCor), University of Sao Paulo Medical School, Sao Paulo, Brazil

**Keywords:** Apex resection, cardiomyocytes neoformation, hemodynamic overload, rats, tissue perfusion

## Abstract

One‐day‐old mice display a brief capacity for heart regeneration after apex resection. We sought to examine this response in a different model and to determine the impact of this early process on long‐term tissue perfusion and overall cardiac function in response to stress. Apical resection of postnatal rats at day 1 (P1) and 7 (P7) rendered 18 ± 1.0% and 16 ± 1.3% loss of cardiac area estimated by magnetic resonance imaging (MRI), respectively (*P* > 0.05). P1 was associated with evidence of cardiac neoformation as indicated by Troponin I and Connexin 43 expression at 21 days postresection, while in the P7 group mainly scar tissue replacement ensued. Interestingly, there was an apparent lack of uniform alignment of newly formed cells in P1, and we detected cardiac tissue hypoperfusion for both groups at 21 and 60 days postresection using SPECT scanning. Direct basal cardiac function at 60 days, when the early lesion is undetectable, was preserved in all groups, whereas under hemodynamic stress the degree of change on LVDEP, Stroke Volume and Stroke Work indicated diminished overall cardiac function in P7 (*P* < 0.05). Furthermore, the End‐Diastolic Pressure–Volume relationship and increased interstitial collagen deposition in P7 is consistent with increased chamber stiffness. Taken together, we provide evidence that early cardiac repair response to apex resection in rats also leads to cardiomyocyte neoformation and is associated to long‐term preservation of cardiac function despite tissue hypoperfusion.

## Abstract (precis)

We provide evidence that 1‐day‐old rats display early repair capacity after apex resection and this response is lost in 1‐week‐old animals similarly described for mice. The repair response is associated with long‐term preservation of overall cardiac function, despite the fact that repair is incomplete and there is tissue hypoperfusion at 21 and 60 day post injury.

## Introduction

The limited postnatal cardiac regenerative capacity in mammalian hearts is one of the major challenges in cardiovascular medicine (Laflamme and Murry 2011; Anversa and Leri 2013; Andersen et al. 2014). Fibrotic scar formation is the predominant repair mechanism as billions of cardiomyocytes are lost during an acute myocardial infarction or other cardiovascular injuries, which is associated with long‐term unfavorable outcomes. In the last decade, several gene/cell‐based approaches were explored to improve the post myocardial infarction (post‐MI) repair response showing promising results in preclinical models. Unfortunately, thus far, no therapeutic scheme has been shown effective in clinical settings. One may speculate that clinical failure is due, at least in part, to the lack of cardiomyocyte replacement, and to the fact that the potentially beneficial effects are restricted to processes such as neoangiovasculogenesis and inhibition of inflammation and collagen deposition, which might be clinically relevant only for specific scenarios (Mummery et al. 2010). The development of strategies to reprogram adult cells into cardiomyocytes ex‐ or in‐vivo together with data showing that the adult heart displays some degree of cardiomyocyte proliferation (Bergmann et al. 2009; Kajstura et al. 2010a,b, 2012; Senyo et al. 2013) and that local cardiac stem cells undergo differentiation (Hsieh et al. 2007; Gonzalez et al. 2008), raise the possibility that targeting these processes may lead or add to more efficient cardiac repair in the adult organ. The latter is consistent with recent data suggesting that neonatal mice hearts retain a robust but transient cardiac regenerative capacity immediately after birth in response to apex resection or ischemic injury (Porrello et al. 2011a,b, 2013; Haubner et al. 2012). The investigation of the underlying healing mechanisms in these models of early heart injury may give insights for novel approaches on effective strategies in adult cardiac repair, which depends on the extracellular matrix remodeling, establishment of adequate tissue vascular supply, and newly formed cardiomyocytes acting as a syncytium.

In the present study, we have two major goals. First, to extend this observation to an another species, the rat model, where a great body of functional knowledge is available regarding acute and chronic consequences of cardiac injury; second, we sought to assess the hitherto unknown long‐term efficacy of cardiac repair through development, specifically to cope with hemodynamic stress during adulthood. The rat is a suitable model for investigating these issues, because there are standard methods to assess quantitatively direct hemodynamics and cardiac tissue perfusion. Our data show that the rat indeed displays a robust early postnatal cardiomyocyte neoformation in response to 18% apex resection, leading to maintenance of overall adult cardiac function under hemodynamic stress despite documented tissue hypoperfusion.

## Materials and Methods

### Apical resection surgery

One‐day‐old (P1) and 7‐day‐old (P7) rats (Wistar strain) underwent heart apex resection. Newborns were anesthetized by hypothermia for 10 min. Following a small skin incision, ventrolateral thoracotomy was cautiously performed at the third intercostal space by dissection of the intercostal muscles. The heart was exposed and the apex of P1 and P7 rats was resected using iridectomy scissors. The thoracic wall incision was closed with 7.0 nonabsorbable silk sutures. For recovery, the neonates were placed on a warmed plate (37°C) under a heat lamp with oxygen flow until fully conscious. Sham surgery was similar, except that resection was omitted. P1 and P7 rats at 21 and 60 days after apical resection surgery were anesthetized intraperitoneally with adjusted dose of xylazine chloride and ketamine chloride (15 mg/kg and 60 mg/kg, respectively; Sespo Industria e Comercio Ltda, Paulinia, SP, Brazil) and hearts were quickly harvested.

Experimental procedures followed the US National Institutes of Health and institutional guidelines for care and use of laboratory animals and were approved by the Institutional Review Board of the University of São Paulo Medical School, Brazil (#285/12).

### Magnetic resonance imaging

Magnetic resonance imaging was performed in a 1.5T MR scanner (Philips Achieva, Amsterdam, the Netherlands), using a four‐channel sense wrist coil. Newborn rats were anesthetized by inhalation with adjusted dose of isoflurane (Isothane, Baxter Healthcare, Guayama, Puerto Rico) in approximately 1 L/min oxygen, and were placed over a 250‐mL saline bag, feet first and in a dorsal decubitus position. A 3‐plane localizer 2‐dimensional fast field echo pulse sequence (2D FFE) provided scout images for heart location and prescription of the next image pulse sequence. Image pulse sequence for acquiring the images used for analysis of the heart and its apex was a high‐resolution 3‐dimensional Turbo Spin‐Echo (TSE) with parallel imaging (SENSE). Parameters were the following: 3D acquisition TSE with SENSE; Prescription plane: coronal; 50 slices; Field‐of‐view (FOV): 50(FH) × 50(RL) × 20(AP) mm; Matrix: 112 × 107 pixels; Voxel size: 0.45 × 0.45 × 0.45 mm or 450 *μ*m; Reconstruction matrix: 448 pixels; Reconstruction voxel: 0.112 mm or 112 *μ*m; Number of Averages (NSA): 6; Turbo factor (TSE): 6; Repetition time (Kajstura et al. 2010a): 155 ms; Echo time (TE): 30 ms; Phase direction: RL; Parallel imaging factor (SENSE factor): 1(FH) and 2(RL). The images were strongly proton‐density weighted. Acquisition time for one 3D volume was 8 min and 51 sec. The rats were scanned before and immediately after surgery, and the ones that did not exhibit a resection of at least 15% of apical area were eliminated.

### Histological analysis

Heart tissues of the euthanized animals, 21 days and 60 days after surgery, were harvested and washed with saline to withdraw blood, and then were embedded in O.C.T. compound (Tissue‐Tek, Sakura, NL) and frozen in liquid nitrogen. Frozen hearts were maintained at −80°C until they were cryosectioned. For histological analysis the organ was cryosectioned on 6 *μ*m.

To perform general histopathological analysis, hearts were cryosectioned at 6‐*μ*m and stained with Hematoxilin–Eosin (HE). Other sections were stained using the Picrosirius method in a 0.1% Sirius Red solution (Direct Red 80, Sigma Aldrich, Saint Louis, MO) in saturated picric acid, followed by rapid washing in running tap water, without any counterstaining for histological evaluation of collagen fibers.

### Picrosirius‐polarization method

Picrosirius‐stained sections were evaluated by polarization microscopy. The Picrosirius‐polarization method (PSP) is a method that allows study of the arrangement and aggregation of collagen fibers due to a normal birefringence exhibited by their molecules disposed in an orderly parallel orientation in the tissues(Borges et al. 2008).

### Immunofluorescence

To assess the formation of new cardiomyocytes, sections were labeled with Troponin I. Slides were labeled for cardiac Troponin I (ab19615, 1:50, Abcam, Cambridge, MA) and Connexin 43 (ab11370, 1:50, Abcam, Cambridge, MA). Alexa fluor 488‐ and 555‐(A11011 and A21428, 1:400, Invitrogen, Life Technologies, Grand Island, NY) conjugated secondary antibodies were used for double staining, and DAPI (D3571, 1:100, Invitrogen, Life Technologies) for nuclei staining.

### Single photon emission tomography/computed tomography

P1, P7 were scanned 21 and 60 days after apical resection surgery in a fully integrated trimodality single photon emission tomography/computed tomography (SPECT/CT) Triumph^™^ scanner manufactured by GE Healthcare (Waukesha, WI). Each rat was anesthetized with 1.5–2.5% isoflurane in 2 L/min oxygen, and injected with approximately 37 MBq [^99m^Tc]Sestamibi in the tailvein. The animal was positioned in the scanner, and SPECT images were acquired 30 min after injection. Each detector head was fit with a 5‐tungsten pinhole 1 mm in diameter. The radius of rotation was set to 4.4 cm, and data were acquired over 128 projection angles (180° for each head), 9Kcts per projection, giving a total acquisition time of approximately 30 min. Projection data were acquired into a 60 × 60 matrix, with pixel size 1.15 mm. Images were reconstructed using OSEM methods with five iterations and eight subsets. Data were analyzed with *π* PMOD 3.4002A software, with interpolation of 1.0 and 3D Gaussian filters. Guided shafts were used with and without a filter to find the cardiac short‐axis, and vertical and horizontal long axis. The semi‐automatic mode was used to guide Bull's‐eye segments, shared it in 17 segments, where five represent the apex area and 12 represent remote area. Data obtained were normalized as a percentage of the cardiac segment of higher activity. A skilled professional was the blinded analyzer of the Bull's‐eye quantification.

### Hemodynamic measurements

Invasive hemodynamic studies were performed to evaluate cardiac performance 60 days after surgery in P1, P7, and sham rats. Each rat was anesthetized intraperitoneally with adjusted dose of urethane chloride (1.5 g/kg; Sigma‐Aldrich). The left femoral vein was accessed to supplement anesthesia, drugs, or saline. A MicroTip pressure–volume catheter (model 1.4 French SPR 839; Millar Instruments, Houston, TX) was then inserted into the right carotid artery and positioned immediately above the aortic valves to monitor aortic blood pressure. After 5 min of arterial blood pressure recording, the catheter was advanced into the left ventricle (LV) cavity for simultaneous and continuous pressure and volume measurements. The right jugular vein was also cannulated, and a 10‐*μ*L bolus of 15% saline was injected to measure parallel conductance. Volume calibration was accomplished by using linear volume conductance regression of the absolute blood volume (four cylindrical chambers containing a specified volume of fresh heparinized rat blood) versus the corresponding signal acquired by the conductance catheter. Data were acquired for computer analysis (PVAN Software, Millar Instruments, Houston, TX) using LabChart 7 Software System (PowerLab, ADInstruments, Bella Vista, NSW, Australia). The following parameters were obtained: heart rate (Senyo et al. 2013), mean arterial pressure (MAP), LV end‐systolic and end‐diastolic pressures (LVESP and LVEDP, respectively), maximal rate of LV pressure rise (+*dP/dt*max) and decline (‐*dP/dt*max), stroke volume (SV), cardiac output, and stroke work (SW). Hemodynamic parameters were determined under basal conditions and during a sudden pressure overload with a vasoconstrictive phenylephrine hydrochloride (PHE; Sigma‐Aldrich) bolus injection (25–75 mg/kg body weight) into the left femoral vein after bilateral vagotomy to prevent changes in heart rate values induced by the baroreflex. PHE doses were adjusted for individual animals to produce comparable elevations in blood pressure (60–80% greater than baseline). The slope of the linear relation between the end‐systolic volumes and pressures (ESPVR) was obtained during transient occlusions of the inferior vena cava in the experimental groups. After measurements, the hearts were quickly harvested and weighed.

### Statistical analysis

Results are presented as mean ± standard error of the mean (SEM). The unpaired Student's‐*t* test was used to compare Troponin I and collagen fiber content in the resected area from P1, P7, and sham groups after 21 and 60 days of apex resection. One‐way ANOVA with post hoc Tukey's test was used to compare interstitial collagen fiber deposition and hemodynamic measurements in the resected groups. Two‐way ANOVA with post hoc Bonferroni's test for repeated measures was used to compare the heart perfusion of resected groups after 21 and 60 days of apical resection surgery. All statistical analyses were performed using GraphPad Prism 5.0 (GraphPad Softwares Inc., La Jolla, CA). A value of *P* ≤ 0.05 was considered to indicate statistically significant differences between tested groups.

## Results

### Quantification of apex resection in neonatal rats by MRI

**Figure 1.**
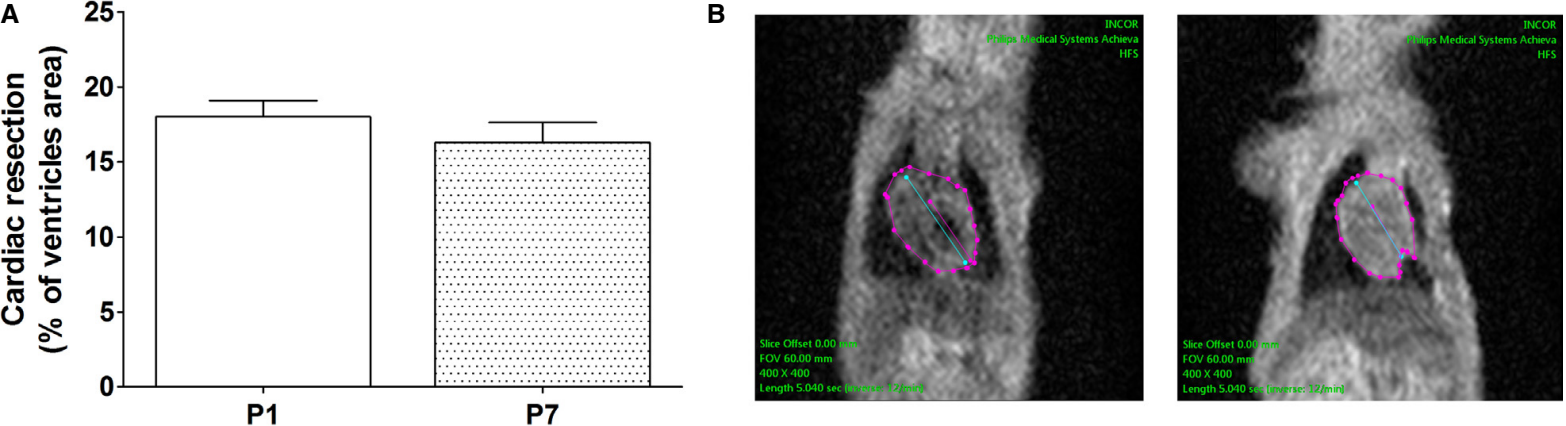
Percentages (mean ± SEM) of ventricles resected (A) in P1 and P7 (*n* = 12 for both); (B) Magnetic resonance images obtained before and immediately after surgery. Dotted lines show the heart area used for resection quantification.

The resected area in 1‐ and 7‐day‐old animals (P1 and P7, respectively) was determined by magnetic resonance imaging (MRI) via the acquisition of a 3‐plane localizer 2‐dimensional fast field echo pulse sequence before and immediately after heart apex resection in neonatal rats (Fig. 1). The average ventricle resection area was similar between P1 and P7 groups [18 ± 1.0% and 16 ± 1.3%, *P* > 0.05, respectively (Fig. 1A)]. Figure 1B displays a representative image of MRI in P1 before and immediately after surgery illustrating the resected portion of the apex area. It is important to emphasize that the survival rate after resection reached 63% and 53% for P1 (*N* = 331) and P7 (*N* = 265), respectively, with most of the losses occurring immediately after the procedure.

### Repaired tissue in P1 is rich in cardiac Troponin I and Connexin 43 expression

**Figure 2.**
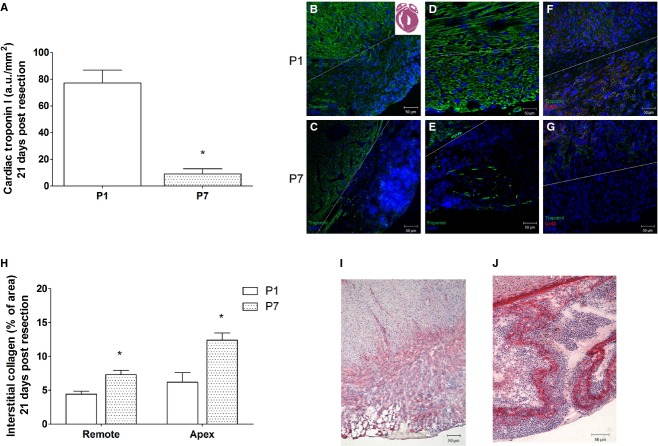
Cardiac Troponin I quantification (a.u./mm^2^), collagen fiber quantification according to Picrosirius red staining (percentage of area) and Connexin 43 labeling into neoformed tissue. (A) Bars indicate labeling of cardiac Troponin I in green per area (mean ± SEM) of P1 (*N* = 6) and P7 (*N* = 7) 21 days after resection surgery. Images indicate the labeling of cardiac Troponin I of (B) P1 (20 × ), (C) P7 (20 × ), (D) P1 (40 × ), (E) P7 (40 × ). Images indicate labeling of Connexin 43 of (F) P1 (20 × ), (G) P7 (20 × ). (H) Bars indicate percentage of apical and remote collagen fibers (mean ± SEM) of P1 (*n* = 8 and 5, respectively) and P7 (*n* = 10 and 7, respectively) 21 days after resection surgery. Images indicate labeling of collagen fibers of (I) P1 (20 × ), (J) P7 (20 × ). *P < 0.05.

To verify whether cardiomyocytes were in the repaired tissue, cardiac Troponin I labeling was performed in samples from both P1 and P7 groups 21 days after apex resection (Fig. 2). Cardiac Troponin I labeling was predominant in P1 versus P7 (8.5‐fold increase), and the labeling was uniformly distributed in P1 compared to a scattered and patched distribution in P7 (P1: 77.3 ± 9.5 a.u./mm² vs. P7: 9.0 ± 3.9 a.u./mm²; *P* ≤ 0.05, Fig. 2A). In addition, the newly formed cells in P1 also expressed Connexin 43 suggesting the presence of mature cardiomyocytes with transmembrane proteins that assemble to form gap junctions (Fig. 2F). Conversely, the patchy Troponin I‐positive scattered cells in P7 hardly expressed Connexin 43 (Fig. 2B–G). Nuclei staining (DAPI) showed predominantly noncardiomyocyte cells (Troponin I negative cells) in repaired apex of P7 compared to P1 (Fig. 2B–E). Fibrosis assay showed that in P1 both the apex and remote areas displayed lower amounts of collagen compared to P7 (P1: 6.2 ± 1.4 vs. P7: 12.4 ± 1.0; apex collagen; P1: 4.4 ± 0.4 vs. P7: 7.3 ± 0.6 remote collagen; Fig. 2H–J; *P* ≤ 0.05).

### Repaired tissues in P1 and P7 display hypoperfusion at 21 and 60 days post apex resection

**Figure 3.**
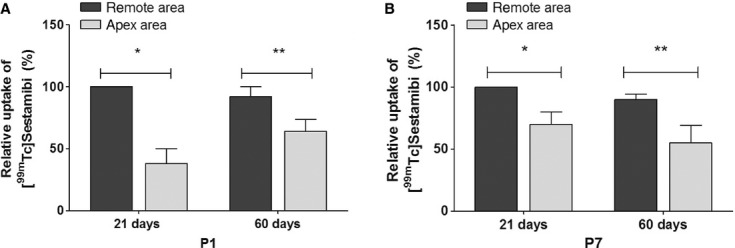
Myocardial perfusion performed by Single Photon Emission Tomography (SPECT) scanning of the heart segments of P1 and P7. Bars indicate relative up take of [^99m^Tc]Sestamibi in the apex area (5 segments) and in the other portions of heart (12 segments) of (A) P1 (*n* = 6) and (B) P7 (*n* = 5). *P < 0.05.

As shown above, at 21 days postresection, there was poor fibrosis deposition and cardiomyocyte formation in the repaired tissue of P1 animals, even though the tissue architecture and the alignment of the cardiac Troponin I‐ and Connexin 43‐positive cells did not appear well preserved (Fig. 2 and data not shown). We then performed SPECT scanning using [^99m^Tc]Sestamibi as a radiopharmaceutical to verify the blood perfusion of the repaired apical tissue. The radiotracer concentrates in the myocardium after systemic administration and the perfusion is proportional to regional blood flow. Figure 3 shows that the same animals from both P1 and P7 displayed reduced uptake of [^99m^Tc]Sestamibi on apex versus the remaining areas of the heart at 21 days (38 ± 12% and 70 ± 10%, respectively) and 60 days postresection (64 ± 9.8% and 55 ± 14.0%, respectively).

### Long‐term overall cardiac function is preserved in the P1 group under hemodynamic stress

**Figure 4.**
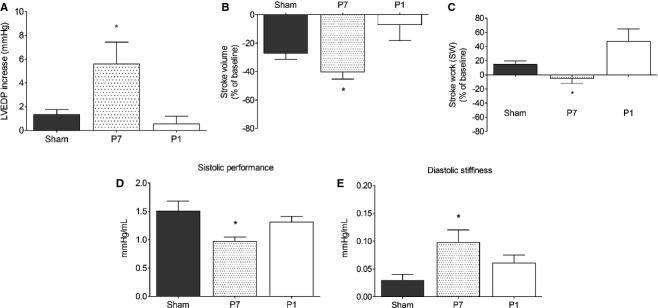
Cardiac response to afterload stress. Bars represent repercussions induced by phenylephrine injection, expressed as % of change from baseline (except for LVEDP, expressed as mmHg elevation over baseline), on the LV end‐diastolic pressure (A) (LVEDP), (B) Stroke Volume, and (C) Stroke Work of sham (*n* = 6), P7 (*n* = 6) and P1 (*n* = 5) groups. Positive or negative values resulted from increase or decrease in the evaluated parameter, respectively. Overall slope values of the ESPVR as an index of (D) systolic performance and EDPVR as an index of (E) diastolic stiffness. *P < 0.05 versus P7.

Considering the characteristics of the repaired tissue and the hypoperfusion in the P1 group, it became essential to assess the overall cardiac function in the adult animals, especially under hemodynamic stress to evaluate the long‐term effectiveness of the repaired myocardium. Cardiac performance was measured directly under basal and pharmacological stress conditions 60 days after the surgical procedure. Interestingly, under basal conditions, overall cardiac function was preserved in adult P1 and P7 apex resected groups compared to sham (Table 1). In contrast, main hemodynamic parameters related to cardiac performance in response to afterload stress were compromised in P7 compared to P1 and sham groups (Fig. 4). Upon pharmacologic stress, changes in LVEDP from basal levels (mmHg) were significantly higher in the P7 group, whereas P1 and shams responded similarly suggesting an ejection deficit in P7 (P7: 5.6 ± 1.8 vs. sham: 1.3 ± 0.4 and P1: 0.6 ± 0.6 mmHg; *P* ≤ 0.05, Fig. 4A). Also, the degree of change on SV from basal levels in response to pressure overload displayed similar behavior, with P7 showing the largest reduction in SV changes compared with P1 and sham (P7: −40.3 ± 4.9 vs. sham: −27.1 ± 4.1; P1 and −6.9 ± 11.4% of change; *P* ≤ 0.05; Fig. 4B). The changes in SW from basal levels, which represents a global index of cardiac performance that depends on both pressure generation and ejection capability during each beat, displayed comparable profile between sham and P1 and diminished in P7 (P7: −4.8 ± 7.2 vs. sham: 15 ± 4.7 and P1: 47 ± 17.8% of change; *P* ≤ 0.05; Fig. 4C). The slope of the linear relation between the End‐Systolic Volume and Pressure (ESPVR) decreased in the P7 group compared to shams and P1 consistent with a decreased systolic performance in P7 (P7: 0.97 ± 0.07 vs. sham: 1.5 ± 0.17 and P1: 1.3 ± 0.1 mmHg; *P* ≤ 0.05; Fig. 4D). Finally, the slope of the end‐diastolic P–V relationship (EDPVR) increased in P7 compared to sham and P1 suggesting increased diastolic stiffness in the P7 group (P7: 0.1 ± 0.02 vs. sham: 0.03 ± 0.01 and P1: 0.06 ± 0.01 mmHg; *P* ≤ 0.05; Fig. 4E).

**Table 1.** Biometric and hemodynamic parameters of sham animals, P7 and P1 under basal conditions.

	SHAM	P7	P1	ANOVA (*P* value)
*Biometry*				
Body weight, g	327 ± 53	306 ± 64	337 ± 28	0.45
Heart/BW, mg/g	0.0039 ± 0.0003	0.0040 ± 0.0004	0.0036 ± 0.0004	0.23
*Hemodynamics*				
HR, beats·min^‐1^	370 ± 12.4	378 ± 15.4	341 ± 20.1	0.29
MAP, mmHg	85 ± 3.07	89 ± 5.8	98 ± 8.6	0.37
LVSP, mmHg	113 ± 5.8	119 ± 4.8	122 ± 9.01	0.48
LVEDP, mmHg	9 ± 1.7	13 ± 4.3	10 ± 0.9	0.58
CO, *μ*L/min	40,600 ± 3489	35,771 ± 6619	41,100 ± 9339	0.80
EF, %	75 ± 7.8	65 ± 3.3	69 ± 14.06	0.47
SW, mmHg/mL	11,439 ± 940	10,383 ± 2265	13,391 ± 2852	0.082
+dP/dtmax, mmHg/sec	10,229 ± 961	10,123 ± 1044	10,674 ± 2233	0.99
−dP/dtmax, mmHg/sec	−6057 ± 567	−6061 ± 520	−5496 ± 456	0.80
SV, *μ*L	110.5 ± 5.3	89.63 ± 12.02	111.4 ± 16.2	0.27
EDV, *μ*L	151 ± 12.5	147 ± 25.5	177 ± 16	0.87

BW, body weight; HR, heart rate; MAP, mean arterial pressure; LVSP, left ventricular (LV) systolic pressure; LVEDP, LV end‐diastolic pressure; CO, cardiac output; EF, ejection fraction; SW, stroke work; +dP/d*t*max and −dP/d*t*max, maximal rate of LV pressure increment and decrement, respectively; SV, stroke volume; EDV, end‐diastolic volume.

Values are means ± SEM.

**Figure 5.**
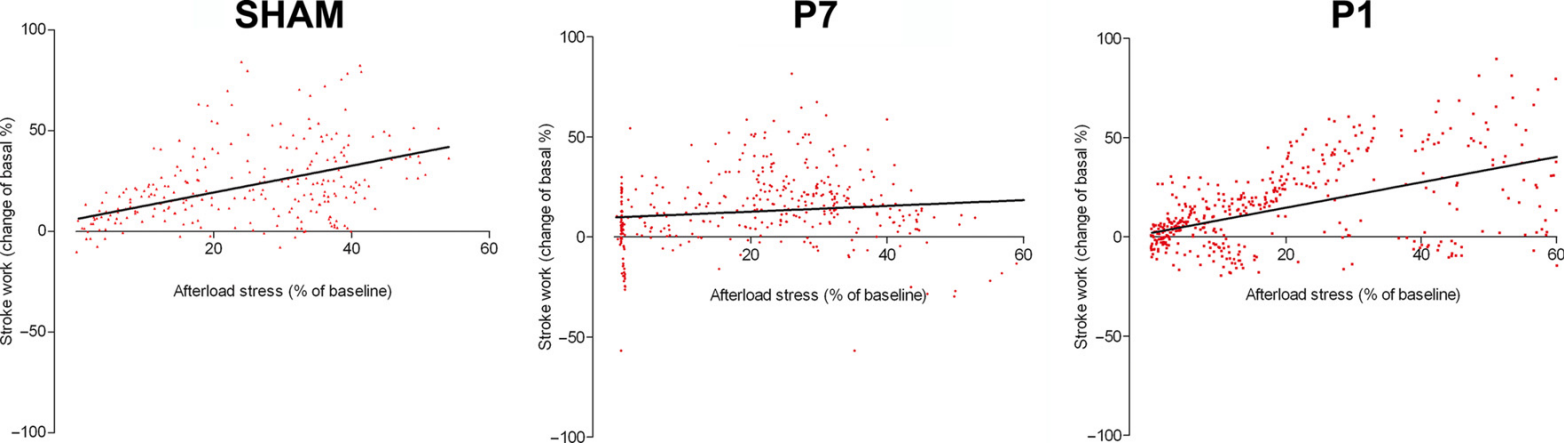
Linear regression curves of the stroke work as a function of increment in systolic pressure. Data are expressed as percentage average change of baseline values for all experimental groups.

In addition, the relationship between the changes in stroke work generation and the increments in systolic pressure (Fig 5) resulted in similar positive correlations in the sham and P1 groups (Pearson's *r*: 0.5; mean slope: 0.66 ± 0.07 and Pearson's *r*: 0.57; mean slope: 0.64 ± 0.04, respectively), whereas the correlation in P7 group was almost flat (Pearson's *r*: 0.13; mean slope: 0.14 ± 0.05). This is in agreement with diminished systolic performance in P7 compared to sham and P1 groups. Other hemodynamic parameters showed no differences between groups under stress in relation to the baseline are shown in Table 2.

**Table 2.** Biometric and hemodynamic parameters of sham animals, P7 and P1 under stress.

	SHAM	P7	P1	ANOVA (*P* value)
*Hemodynamics*				
HR	−2.21 ± 0.28	−4.13 ± 0.77	7.0 ± 7.3	0.24
LVSP	47.2 ± 1.3	53.7 ± 2.2	53 ± 1.3	0.28
CO	−17.5 ± 12.5	−58.9 ± 9.8	−25.8 ± 11.6	0.06
EF	−35.72 ± 6.6	−51.33 ± 6.8	−30.44 ± 9.5	0.17
+dP/dtmax	51.9 ± 8.7	42.6 ± 5.3	45.1 ± 6.4	0.62
−dP/dtmax	81.2 ± 11.5	92 ± 12.5	92.7 ± 17.6	0.79

HR, heart rate; LVSP, left ventricular (LV) systolic pressure; CO, cardiac output; EF, ejection fraction; +dP/d*t*max and −dP/d*t*max, maximal rate of LV pressure increment and decrement, respectively.

Values are means ± SEM and indicate the percentage of change from baseline.

**Figure 6.**
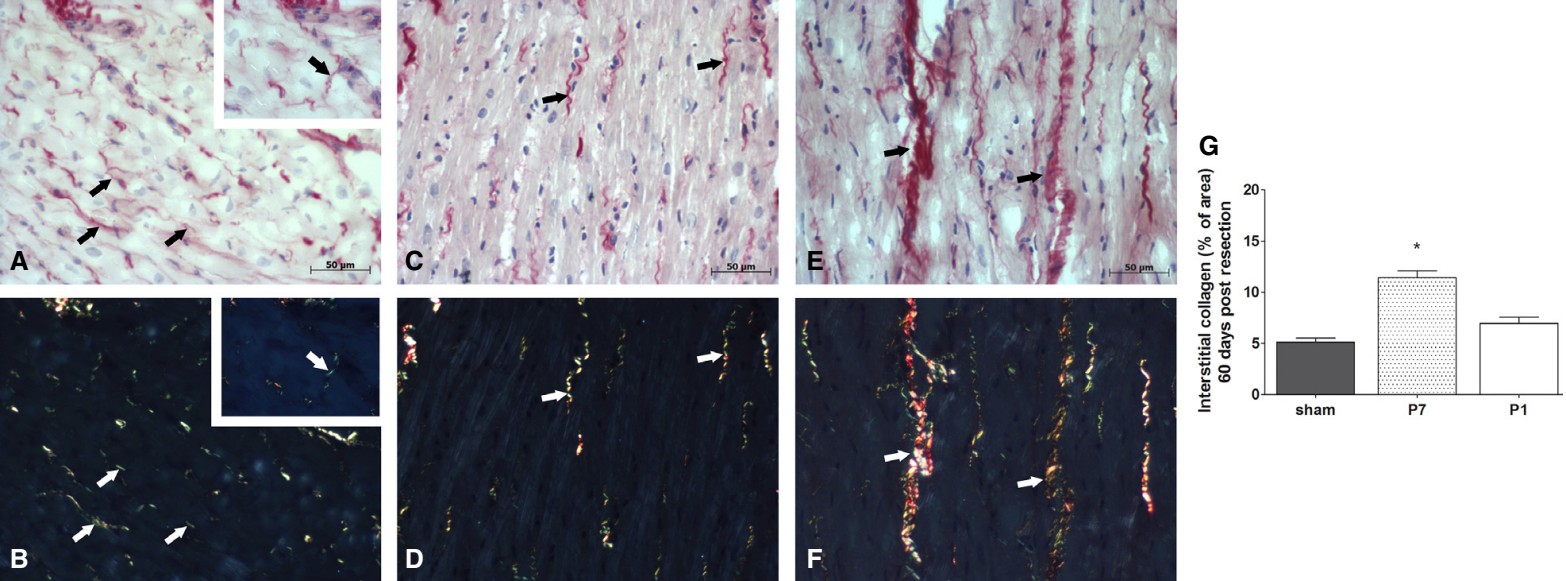
Interstitial collagen fiber quantification into heart coronal sections from P1 and P7 60 days of resection surgery according the Picrosirius method. Images indicate Picrosirius–hematoxylin (A, C, and E) and Picrosirius–hematoxylin under polarized light (B, D, and F). (G) Bars indicate mean percentage of collagen fibers (mean ± SEM) of sham (*n* = 6), P1 (*n* = 5) and P7 (*n* = 6). *P < 0.05 versus P7.

These findings were consistent with increased interstitial collagen fiber deposition (fibrosis) found in adult hearts from P7 compared to P1 and sham groups (P7: 11.4 ± 0.64 vs. sham: 5.1 ± 0.37 and P1: 6.9 ± 0.6; *P* ≤ 0.05; Fig. 6G). Note that these findings are consistent with the results observed at 21‐days postresection shown in Fig. 2H–J. The analysis with the Picrosirius‐polarization method showed that cardiomyocytes from sham and P1 groups at 60 days postresection were surrounded by thin, pale (weakly birefringent), greenish collagen fibers (Fig. 6 A–D). In the P1 group, few cardiomyocytes were surrounded by thick yellow fibers with increased birefringence (Fig. 6D). On the other hand, the ventricular muscle tissue in the P7 group exhibited many cardiomyocytes surrounded by thick, bright (strongly birefringent) yellow, or red collagen fibers (Fig. 6E and F).

## Discussion

The main findings of the present work are that 1. early neonatal rat heart injury (P1) is associated with Troponin I‐ and Connexin 43‐positive cells, and similarly to mice (Porrello et al. 2011b) this response is time‐restricted, since it is lost in 7‐day‐old rats, and 2. this type of repair may provide long‐term preservation of cardiac function, even under hemodynamic stress, despite the fact that the repair response is accompanied by tissue hypoperfusion. We used a reliable method to estimate the percentage of apex resection via magnetic resonance image scanning as an excluding criterion to avoid differences that might compromise comparisons and the claimed conclusions. Only rats exhibiting 15% or more of apical resected area were included in the study. Twenty‐one days postresection, Troponin I‐positive cells were the predominant cells in the repaired area of the apex in the P1 group due to the small, but noticeable, collagen fibers along the repaired area, demonstrating that this is not a complete regenerative process. In contrast, when the injury was produced a few days later, in P7 animals, the reparative process was primarily via collagen fiber deposition and scar formation, even though small clusters of cardiac Troponin I‐positive cells were seen in the repaired apex (Fig. 2). Our findings indicate that tissue restoration is not complete, but it is more comprehensive in 1‐ versus 7‐day‐old rats. In addition, there is apparent gross tissue architecture disarrangement in the P1 group as indicated by the lack of uniform alignment of the cardiac Troponin I‐ and Connexin 43‐positive cells (Figs. 2,6). These findings appear less robust than the ones reported in the original publication using 15% apex resection in mice (Porrello et al. 2011b) and contrast with the complete lack of cardiac repair reported by Andersen et al. in mice submitted to 10% apex resection (Andersen et al. 2014). One may speculate that the neoformation process is incomplete, because the majority of cardiomyocytes exit from the cell cycle early after birth (Li et al. 1996; Pasumarthi and Field 2002; Ahuja et al. 2007), and the remaining ones that hold this potential for the first few hours after birth might not be sufficient in number to provide complete restoration of the injured tissue. These observations prompted us to explore whether the vascular supply was compromised and the influence of the early repair on later overall cardiac function during adulthood. It is important to bear in mind that the relative injured área (between 16–18%) at the beginning of the protocol is significant, but at 60‐day‐old animals, it is most likely irrelevant due to the large change in heart size between newborns and adult animals. Thus, the long‐term consequences observed may reflect differences in the initial reparative processes triggered in 1‐ or 7‐day‐old rats, and how these events contributed over time to minimize the initial damage while the organ was still growing. In this regard, the data from the tissue perfusion assessments, performed twice in each animal at 21 and 60 days post injury, are presented in relation to the viable remote tissue at each data point in all groups to minimize bias associated with the significant differences in heart weight at 21‐ and 60‐day‐old animals. This method of analysis is used to evaluate global perfusion in myocardial infarction both in experimental and clinical studies (Acton et al. 2006; de Oliveira et al. 2013). Since fibrosis replaces viable muscle tissue, SPECT imaging with [^99m^Tc]Sestamibi is used clinically to estimate the infarct size, because the collagen area lacks tracer uptake (Gibbons 2011). The data analysis was performed blinded using 17 segments of the whole heart by Bulls‐eye imaging (Nuyts et al. 1989) from the apex area (five segments) and from remote area (12 segments) of the same animals at 21 and 60 days after surgery. Indeed, SPECT scanning revealed an imbalance in the vascular supply in the healed tissue of both P1 and P7 groups at 21 and 60 days after surgery. In P7 group, the lack of cardiomyocytes and potential inadequate neoangiovasculogenesis are consistent with the lower tracer uptake. In contrast, the hypoperfusion in P1 was not anticipated despite scarce collagen deposition in the regenerated area. One may speculate that in addition to inadequate blood supply, the neoformed cardiomyocytes may also display functional metabolic abnormalities.

The echocardiographic analysis under basal hemodynamic conditions has traditionally been the method of choice to evaluate cardiac function outcomes in gene/cell therapy approaches post‐MI. However, we have previously demonstrated that rats that undergo experimental MI affecting a large (35–45%) area of the left ventricle indeed display normal hemodynamic parameters assessed by both noninvasive as well as direct intracardiac pressure/volume determination values. Under these conditions, the rodent, unlike humans, will display compromised cardiac function only under pharmacologic stress (Nakamuta et al. 2009; Danoviz et al. 2010; Goncalves et al. 2010; dos Santos et al. 2010). Indeed, the results show that under basal conditions, cardiac global parameters in adult animals were unchanged in all groups using direct hemodynamic assessment (Table 1). In contrast, upon pharmacologic afterload stress the cardiac response was compromised in the P7 and preserved in the P1 group (Figs. 4–5). The preservation of cardiac function in P1, despite apex hypoperfusion, may occur due to a favorable combination of the replacement of functional cardiomyocytes and the absence of significant and rigid fibrotic tissue in repaired apex with no significant compensatory remodeling of the heart. On the other hand, lack of early cardiomyocyte formation and fibrosis scar in P7 led to unfavorable outcomes after phenylephrine administration, such as increased LVEDP, revealing ejection deficit, as commonly shown in myocardial infarction accompanied by elevated LVEDP (Iskandrian et al. 1981). This is consistent with the diminished ESPVR, indicating decreased systolic performance in the P7 group, which also displayed an increased slope in the end‐diastolic P–V relationship, suggesting increased chamber stiffness, correlated with increased interstitial collagen fibers in P7 after 60 days.

The findings of the present study give support to the idea that cardiac repair occurs transiently after birth. The repair response, albeit not complete and accompanied by tissue hypoperfusion, rendered the tissue functional even during stress in adult rats, long after the initial events took place. These results are not readily translated to adult cardiac repair strategies, but indicate the usefulness of the model to gain insight into postnatal mechanisms of cardiac repair. Magnetic resonance imaging was performed immediately before and after injury to ensure that 16–18% of the LV from minute newborn rats indeed is excised despite the fact that the interior of the LV chamber is exposed. If the chamber is not properly sealed, as occurs in few animals, immediate death ensues due to large blood losses in the first few heartbeats after injury and this specific issues may be further explored to reconcile inconsistent findings using variations of this model.

Taken together, we provide, for the first time to rat, evidence that one‐day‐old animals display early repair capacity after apex resection and this response is lost in one‐week‐old animals similarly described for mice. The repair response is associated with long‐term preservation of overall cardiac function, despite the fact that repair is incomplete and there is tissue hypoperfusion at 21 and 60 day post injury. These findings underscore the complexity of the early events associated with the cardiac healing and long‐term impact that they may have on preservation cardiac function.

## Acknowledgments

We thank Monica Nunes, Simone Fernandes, Julio Soares, Prof. Carlos Buschpiegel, Dr. Luciano de Figueiredo Borges, Dr. Fábio N Marques, Josefina Silva, Dr. Marco Antônio de Oliveira, and Dr. Vinicius Bassaneze for the assistance in animal manipulation, MRI performance, *PSP* analysis, SPECT scanning, and data and image analysis.

## Conflict of Interest

None declared.

## References

[b1] Acton P. D. Thomas D. Zhou R. 2006 Quantitative imaging of myocardial infarct in rats with high resolution pinhole SPECT. Int. J. Cardiovasc. Imaging; 22:429-434. 16518671 10.1007/s10554-005-9046-7 PMC2835992

[b2] Ahuja P. Sdek P. MacLellan W. R. 2007 Cardiac myocyte cell cycle control in development, disease, and regeneration. Physiol. Rev.; 87:521-544. 17429040 10.1152/physrev.00032.2006 PMC2708177

[b3] Andersen D. Ganesalingam S. Jensen C. H. Sheikh S. P. 2014 Do neonatal mouse hearts regenerate following heart apex resection? Stem Cell Rep.; 2:406-413. 10.1016/j.stemcr.2014.02.008 PMC3986579 24749066

[b4] Anversa P. Leri A. 2013 Innate regeneration in the aging heart: healing from within. Mayo Clin. Proc.; 88:13 10.1016/j.mayocp.2013.04.001 PMC3936323 23910414

[b5] Bergmann O. Bhardwaj R. D. Bernard S. Zdunek S. Barnabe‐Heider F. Walsh S. 2009 Evidence for cardiomyocyte renewal in humans. Science; 324:98-102. 19342590 10.1126/science.1164680 PMC2991140

[b6] Borges L. F. J. R. Dias R. R. Stolf N. A. G. Michel J. B. Gutierrez P. S. 2008 Collagen is reduced and disrupted in human aneurysms and dissections of ascending aorta. Human Pathol.; 39:437-443. 18261628 10.1016/j.humpath.2007.08.003

[b7] Danoviz M. E. Nakamuta J. S. Marques F. L. dos Santos L. Alvarenga E. C. dos Santos A. A. 2010 Rat adipose tissue‐derived stem cells transplantation attenuates cardiac dysfunction post infarction and biopolymers enhance cell retention. PLoS One; 5:9 10.1371/journal.pone.0012077 PMC2919414 20711471

[b8] Gibbons R. J. 2011 Tc‐99 m SPECT sestamibi for the measurement of infarct size. J. Cardiovasc. Pharmacol. Ther.; 16:321-331. 21821535 10.1177/1074248411414906

[b9] Goncalves G. A. Vassallo P. F. dos Santos L. Schettert I. T. Nakamuta J. S. Becker C. 2010 Intramyocardial transplantation of fibroblasts expressing vascular endothelial growth factor attenuates cardiac dysfunction. Gene Ther.; 17:305-314. 20010629 10.1038/gt.2009.146

[b10] Gonzalez A. Rota M. Nurzynska D. Misao Y. Tillmanns J. Ojaimi C. 2008 Activation of cardiac progenitor cells reverses the failing heart senescent phenotype and prolongs lifespan. Circ. Res.; 102:597-606. 18202313 10.1161/CIRCRESAHA.107.165464

[b11] Haubner B. J. Adamowicz‐Brice M. Khadayate S. Tiefenthaler V. Metzler B. Aitman T. 2012 Complete cardiac regeneration in a mouse model of myocardial infarction. Aging (Albany NY); 4:966-977. 23425860 10.18632/aging.100526 PMC3615162

[b12] Hsieh P. C. Segers V. F. Davis M. E. MacGillivray C. Gannon J. Molkentin J. D. 2007 Evidence from a genetic fate‐mapping study that stem cells refresh adult mammalian cardiomyocytes after injury. Nat. Med.; 13:970-974. 17660827 10.1038/nm1618 PMC2754571

[b13] Iskandrian A. S. Segal B. L. Hakki A. H. 1981 Left ventricular end‐diastolic pressure in evaluating left ventricular function. Clin. Cardiol.; 4:28-33. 7226588 10.1002/clc.4960040107

[b14] Kajstura J. Gurusamy N. Ogorek B. Goichberg P. Clavo‐Rondon C. Hosoda T. 2010a Myocyte turnover in the aging human heart. Circ. Res.; 107:1374-1386. 21088285 10.1161/CIRCRESAHA.110.231498

[b15] Kajstura J. Urbanek K. Perl S. Hosoda T. Zheng H. Ogorek B. 2010b Cardiomyogenesis in the adult human heart. Circ. Res.; 107:305-315. 20522802 10.1161/CIRCRESAHA.110.223024 PMC2987602

[b16] Kajstura J. Rota M. Cappetta D. Ogorek B. Arranto C. Bai Y. 2012 Cardiomyogenesis in the aging and failing human heart. Circulation; 126:1869-1881. 22955965 10.1161/CIRCULATIONAHA.112.118380 PMC3477474

[b17] Laflamme M. A. Murry C. E. 2011 Heart regeneration. Nature; 473:326-335. 21593865 10.1038/nature10147 PMC4091722

[b18] Li F. Wang X. Capasso J. M. Gerdes A. M. 1996 Rapid transition of cardiac myocytes from hyperplasia to hypertrophy during postnatal development. J. Mol. Cell. Cardiol.; 28:1737-1746. 8877783 10.1006/jmcc.1996.0163

[b19] Mummery C. L. Davis R. P. Krieger J. E. 2010 Challenges in using stem cells for cardiac repair. Sci. Transl. Med.‐:1-5. 10.1126/scitranslmed.3000558 20393186

[b20] Nakamuta J. S. Danoviz M. E. Marques F. L. dos Santos L. Becker C. Goncalves G. A. 2009 Cell therapy attenuates cardiac dysfunction post myocardial infarction: effect of timing, routes of injection and a fibrin scaffold. PLoS One; 4:e6005 19547700 10.1371/journal.pone.0006005 PMC2695782

[b21] Nuyts J. Mortelmans L. Suetens P. Oosterlinck A. de Rou M. 1989 Model‐based quantification of myocardial perfusion images from SPECT. J. Nucl. Med.; 30:1992-2001. 2585101

[b22] de Oliveira L. F. Mejia J. de Carvalho E. E. Lataro R. M. Frassetto S. N. Fazan R. Jr 2013 Myocardial infarction area quantification using high‐resolution SPECT images in rats. Arq. Bras. Cardiol.; 101:59-67. 23917507 10.5935/abc.20130110 PMC3998176

[b23] Porrello E. R. Johnson B. A. Aurora A. B. Simpson E. Nam Y. J. Matkovich S. J. 2011a MiR‐15 family regulates postnatal mitotic arrest of cardiomyocytes. Circ. Res.; 109:670-679. 21778430 10.1161/CIRCRESAHA.111.248880 PMC3167208

[b24] Porrello E. R. Mahmoud A. I. Simpson E. Hill J. A. Richardson J. A. Olson E. N. 2011b Transient regenerative potential of the neonatal mouse heart. Science; 331:1078-1080. 21350179 10.1126/science.1200708 PMC3099478

[b25] Porrello E. R. Mahmoud A. I. Simpson E. Johnson B. A. Grinsfelder D. Canseco D. 2013 Regulation of neonatal and adult mammalian heart regeneration by the miR‐15 family. Proc. Natl Acad. Sci. USA; 110:187-192. 23248315 10.1073/pnas.1208863110 PMC3538265

[b26] Pasumarthi K. B. S. Field L. J. 2002 Cardiomyocyte cell cycle regulation. Circulat. Res.; 90:1044-1054. 12039793 10.1161/01.res.0000020201.44772.67

[b27] dos Santos L. Antonio E. L. Souza A. F. M. Tucci P. J. F. 2010 Use of afterload hemodynamic stress as a practical method for assessing cardiac performance in rats with heart failure. Can. J. Physiol. Pharmacol.; 88:724-732. 20651820 10.1139/y10-062

[b28] Senyo S. E. Steinhauser M. L. Pizzimenti C. L. Yang V. K. Cai L. Wang M. 2013 Mammalian heart renewal by pre‐existing cardiomyocytes. Nature; 493:433-437. 23222518 10.1038/nature11682 PMC3548046

